# Visual feedback influences the consistency of the locomotor pattern in Asian elephants (*Elephas maximus*)

**DOI:** 10.1098/rsbl.2023.0260

**Published:** 2023-09-27

**Authors:** Max J. Kurz, John R. Hutchinson

**Affiliations:** ^1^ Institute for Human Neuroscience, Boys Town National Research Hospital, 14090 Mother Teresa Lane, Boys Town, NE 68010, USA; ^2^ Structure and Motion Laboratory, Department of Comparative Biomedical Sciences, The Royal Veterinary College, University of London, Hatfield, Hertfordshire AL9 7TA, UK

**Keywords:** biomechanics, variability, gait analysis, walking, stability, Proboscidea

## Abstract

Elephants are atypical of most quadrupeds in that they maintain the same lateral sequence footfall pattern across all locomotor speeds. It has been speculated that the preservation of the footfall patterns is necessary to maintain a statically stable support polygon. This should be a particularly important constraint in large, relatively slow animals. This suggests that elephants must rely on available sensory feedback mechanisms to actively control their massive pillar-like limbs for proper foot placement and sequencing. How the nervous system of elephants integrates the available sensory information for a stable gait is unknown. Here we explored the role that visual feedback plays in the control of the locomotor pattern in Asian elephants. Four Asian elephants (*Elephas maximus)* walked with and without a blindfold as we measured their stride time intervals. Coefficient of variation was used to assess changes in the overall variability of the stride time intervals, while approximate entropy was used to measure the stride-to-stride consistency of the time intervals. We show that visual feedback plays a role in the stride-to-stride consistency of the locomotor pattern in Asian elephants. These results suggest that elephants use visual feedback to correct and maintain proper sequencing of the limbs during locomotion.

## 1. Introduction

Elephants are the largest terrestrial animals and thereby exemplify the extreme influence of gravitational and inertial forces on locomotor performance. Compared to other quadrupeds, elephants are atypical in that they preserve lateral sequencing of their limbs at all locomotor speeds [1,2]. It has been speculated that this limb sequencing may help to reduce the possibility of ipsilateral limb interference [2–5] and to maintain a statically stable base of support [6]. These features would be important to prevent a fall when moving at high speeds, and when negotiating uneven or difficult terrain. Elephants are relatively slow; [1] they do not reach maximal speeds greater than approximately 7 m s^−1^, whereas smaller animals tend to be relatively faster [7,8]. Static stability may be more crucial in slower animals such as elephants [9], although this is debatable (e.g. turtles) [10,11]. In large land animals, falls can easily be fatal [12–14]. Although there has been a thorough series of studies of the biomechanics of elephant locomotion, none have examined how the nervous system of elephants controls their locomotor pattern or responds to perturbations [1,2,15–17] (table 1).

**Table 1.** Results of statistical analyses for experimental conditions of ‘vision’ and ‘no vision’. ApEN = approximate entropy; CV = coefficient of variation for the stride time interval; s.d. = standard deviation of the stride time interval; s.e.m. = standard error of the mean; ST = stride time interval. *T*-tests: ApEN = 0.0267; CV = 0.405; s.d. = 0.204; ST = 0.404.

	vision	no vision	vision	no vision	vision	no vision	vision	no vision
subject number	ApEn	ApEn	ST	ST	s.d.	s.d.	CV	CV
1	1.237	1.520	1.612	1.590	0.0761	0.114	4.716	4.862
2	1.716	1.928	2.057	1.878	0.0836	0.128	4.066	6.796
3	1.814	1.900	1.931	1.973	0.108	0.107	5.580	5.409
4	1.506	1.637	1.677	1.654	0.0740	0.0724	4.411	4.376
mean	1.568	1.746	1.812	1.774	0.0854	0.105	4.693	5.361
s.d.	0.256	0.200	0.210	0.181	0.0155	0.0235	0.648	1.046
s.e.m.	0.128	0.100	0.105	0.0906	0.0077	0.0118	0.324	0.523

For each stride in an elephant, the neuromuscular system must accelerate and decelerate the limbs in a coordinated fashion. Potentially, the massive pillar-like limbs should require precise active neuromuscular control to overcome the limbs' inertia for proper foot placement and inter-limb coordination. Errors in the control of the limbs’ performance would result in slight variations in the locomotor pattern, which could affect the overall sequencing of the limbs if errors became sufficiently large. Greater variability in the temporal and spatial kinematics of the footfall patterns in humans are related to an impaired ability to use the available sensory feedback to correct errors in the walking pattern [18,19]. Although variability measures have provided a wealth of information about how humans control locomotion, these measures have not been widely applied for the exploration of the motor control of animal locomotion [20].

The visual stream from the occipital cortices provides pertinent information to the parietal cortices for calculating egocentric orientation, planning of motor action, and correcting the limbs' performance during locomotion [21,22]. Indeed, removal of vision in humans increases the variability or errors in foot placement during walking [23,24]. The influence of visual feedback on locomotor control in animals has not received much attention in the comparative biomechanics community. Visual feedback may play an important role in elephant locomotion because an incorrect foot placement and sequencing of the limbs could result in a fall. It is alternatively possible that visual feedback may play less of a role because elephants tend to be more active at night [25]. Furthermore, the large mass and hence inertia of elephants may render them relatively stable with little need for stringent higher-level control except when faced with major perturbations. However in such a large, relatively slow animal, passive dynamic properties (i.e. ‘preflexes’) might be less effective than active control [26–28]. Thus elephants may have greater reliance on kinesthetic and somatosensory feedback to correct their walking pattern. This speculation is reinforced by evidence that elephants and other large animals have longer nerve conduction delays and thus slower responsiveness as well as diminished sensorimotor resolution because of the scaling of axonal conduction velocity and diameter [9]. Thus some mechanisms, including vision, for improving sensorimotor responsiveness and resolution may become more critical in elephants. Although these notions are all plausible (despite conflicting to some degree), no efforts have been made to reveal what specific kinds of sensory feedback may be important for the control of elephant locomotion.

Here we explored the role vision plays in controlling elephant locomotor patterns. We measured the stride time (i.e. duration of a complete cycle of footfalls) intervals of their locomotion with and without vision. The ‘no vision’ condition was accomplished by having the elephants walk while wearing a blindfold. Variability measures were used to assess the ability of the elephants to correct and maintain proper sequencing of the limbs during locomotion. The coefficient of variation (CV) and approximate entropy (ApEn) were used to quantify the overall variability of the stride time intervals and the stride-to-stride consistency of the time intervals, respectively [29,30]. ApEn is a regularity statistic that evaluates the likelihood that similar patterns in the time series will be present at a later time period. ApEN is different from the CV in that it evaluates the time-evolving variations that are present in the time series, whereas the CV only evaluates the overall magnitude of the variations [31]. These variability measures were used to test the hypothesis that the overall variability and consistency of the stride time intervals would be disturbed when the elephants walked without vision. Support for this hypothesis would provide novel insights into the potential neuromechanics of how elephants control their limb spatiotemporal kinematics during gait.

## 2. Material and methods

**Figure 1.**
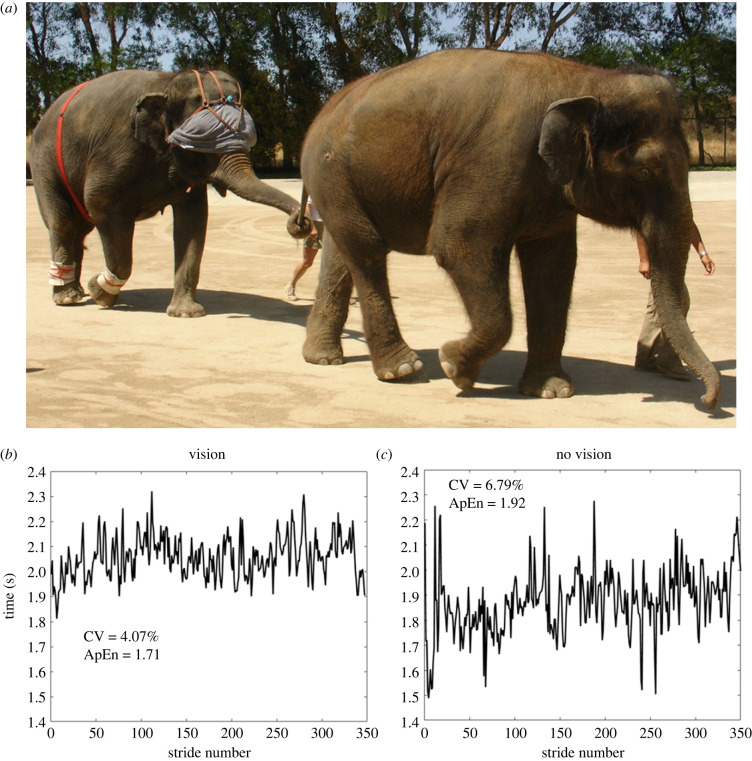
Depiction of the experimental set-up for the 'no vision' condition where the trailing elephant was walking with a blindfold while instrumented and holding on to the tail of the lead elephant. A lead elephant was necessary for maintaining a sustained and consistent walking speed. Representative stride time interval time series are also shown for an elephant while walking with vision (*b*) and without vision (*c*). As shown, the stride time intervals visually appear to have more overall variability and are less consistent during the 'no vision' condition. These observations are confirmed by the coefficient of variation (CV) and approximate entropy (ApEN) values that show there was more variability overall in the stride time intervals, and the stride-to-stride variations in the time intervals were less consistent without vision.

Four healthy adult female Asian elephants (*Elephas maximus)* participated in this study (age = 29.5 ± 11.6 years; mass = 3467.4 ± 381 kg; hip height = 1.73 ± 0.5 m). The experiments were done at the Have Trunk Will Travel facility (Perris, CA, USA). Elephants were given rest, shade, water, food, positive reinforcement and companionship ad libitum to maximize comfort and minimize stress during the experiments. No animals were harmed for this study. For safety reasons, only slow walking speeds were investigated. Each elephant was equipped (figure 1*a*) with a uniaxial accelerometer (ADXL150, Analogue Devices, Norwood, MA, USA) that was mounted on the dorsal side of the toenail of the right hindlimb using melted glue (the right forelimb had the same apparatus, but we assumed symmetry here and did not use those data). The accelerometer signals were collected with a custom portable data logger at 44.1 kHz. The data logger was mounted on the lateral aspect of the distal portion of the lower limb with elastic bandages. A global positioning system (GPS; BTGPS II, EMTAC Technology, USA), used to measure locomotor speed, was strapped around the elephant's torso. Similar data collection methods have successfully been used for evaluating the locomotor patterns of horses and elephants [32,33].

Elephants walked back and forth along a 90 m outdoor walkway while holding the tail of a lead elephant that was coached by a trainer. A lead elephant was used to ensure that the elephants were walking at a consistent and similar speed between experimental conditions. This was necessary because blindfolded elephants could not maintain a steady walking speed or direction, and the guidance of a friendly elephant helped to assuage any anxiety in these sensitive animals. The elephants completed a minimum of 10 walking trials with and without vision. The experimental conditions were presented in a random order. The no vision condition was accomplished by having the elephant wear a blindfold made of heavy black cloth that was tucked into a head harness. It was very evident that the elephants could not see while wearing the blindfold. When the trainers would indicate for them to grab the tail of the lead elephant, they would often miss or have to feel around with their trunk to find the other elephant's tail. Elephants, however, did not extensively use their trunks to navigate while walking with no vision.

The GPS data were downloaded using the CRUX II GPS software (EMTAC Technology, USA), and the average walking speed was calculated for the respective trials. The data from the foot accelerometer were re-sampled to 256 Hz, and then the foot touchdowns were identified with custom software [33]. Segments of the walking conditions where the elephant was turning around at the end of the walkway were removed, and the remaining data sections were concatenated together to create a continuous time series. A median filter was used to eliminate the stride time intervals that were greater than three standard deviations higher or lower than the median value [30,34].

The CV was used to calculate the percentage of overall variability in the stride time interval time series using equation 2.1, where s.d. is the standard deviation and *M* is the mean of the time series. A larger CV indicates more overall variability in the stride interval time series.

2.1 $$\mathrm{CV}=\left(\frac{s.d.}{M}\right)\times 100.$$

The ApEn was calculated using equation (2.2), where *m* and *m* + 1 are the stride time intervals compared, *r* is the similarity criterion, and *C_m_*(*r*) is the number of stride time intervals that are found to be similar in the time series [29,30].

2.2 $$ApEn(m,r)=\ln\left[\frac{C_{m}(r)}{C_{m+1}(r)}\right].$$

The stride time intervals were iteratively compared across the time series and were considered similar if the difference was less than a fixed similarity criterion of *r* = 0.02. In principle, selecting an *r* that is too small will limit the number of stride times that are similar, while selecting an *r* that is too large can result in too many self-similar matches. Multiple methods have been proposed for selecting a similarity criterion [30,35–37]. Similar to prior investigations, the similarity criterion was determined through a parameter search until consistent and stable ApEN values were achieved [30,38]. A small ApEN value indicates greater stride-to-stride consistency in the selected time intervals. Conversely, a large ApEN value indicates lesser consistency in selecting the stride time intervals from one stride to the next.

Separate two-tailed dependent *t*-tests at a 0.05 alpha level were used to discern if the CV and ApEN values were significantly different when the elephants walked with and without vision. We additionally calculated the Cohen's *d* values to determine the effect size of any differences noted in the elephant's gait while walking without vision. Cohen's *d* values less than 0.2 are small effects, 0.5 are medium, whereas values greater than 0.8 are large effects visible to the observer [39]. Results are displayed as the mean ± standard error of the mean.

## 3. Results and discussion

The average walking speeds for the vision and 'no vision' conditions were 0.34 ± 0.05 m s^−1^ and 0.33 ± 0.06 m s^−1^, respectively. In addition, the average stride times for the vision and 'no vision' conditions were 1.82 ± 0.1 s, and 1.77 ± 0.1 s, respectively. Together these results indicated that the elephants walked at a similar walking speed for each condition but appeared to have a slightly altered mean stride time interval when vision was removed. The average stride time intervals were not significantly different between conditions (*p* > 0.05).

Representative stride time interval series from the respective conditions are shown in figure 1. Qualitatively, it is apparent that the stride time intervals were less variable during the vision condition (figure 1*b*) compared to the 'no vision' condition (figure 1*c*). The higher CV and ApEn values for this exemplary elephant confirm this observation: overall there was more variability and less consistency in the stride time intervals when vision was removed.

**Figure 2.**
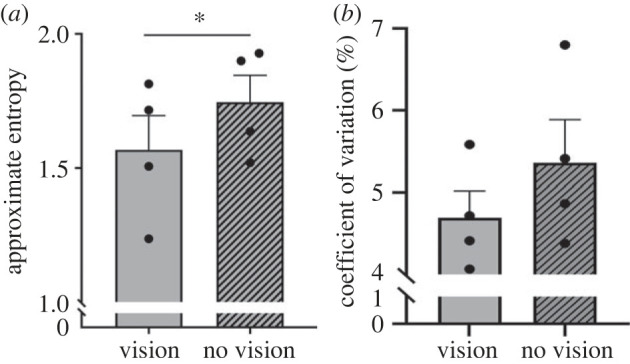
(*a*) Approximate entropy (ApEN) and (*b*) coefficient of variation values of the stride time interval time series when elephants walked with and without vision. The ApEN stride time intervals were statistically less consistent when the elephants walked without vision. The effect size was *d* = 1.92, indicating that the difference in the consistency of the locomotive pattern when vision was removed was large and discernible. Data are presented as mean ± s.e.m. with individual participant data points. *p ≤ 0.05.

There was no significant difference in the CV (vision = 4.69 ± 0.32%; 'no vision' = 5.36 ± 0.52%; *p* = 0.40), although elevated during the 'no vison' condition, suggesting that the overall variability of the stride time series was similar between the respective conditions. However, there was a significant difference between the ApEn values (vision = 1.57 ± 0.13; 'no vision' = 1.75 ± 0.10; *p* = 0.02). This suggests that the stride-to-stride variations in the time intervals were less consistent when the elephants walked without vision (figure 2). The Cohen's *d* value for this finding was large (*d* = 1.92), indicating that the removal of visual feedback had a noticeable influence on the gait consistency of the elephants.

Our results are the first to show that visual feedback might play an important role in the control of an elephant's locomotor patterns. When vision was removed, the elephants' nervous systems might have had less certainty in selecting an appropriate stride time interval. This effectively might result in greater variations or errors in the sequencing of the limb movement patterns. This increased stride-to-stride locomotor variability suggests that vision is important for controlling locomotion in elephants. Visual feedback should provide elephants with important information about the body's position relative to the environment, and changes in terrain. This feedback is essential for controlling of the limb sequencing, especially salient considering the highly variable terrestrial environments that elephants often encounter. In addition to the visual feedback that our study indicates is important, the nervous system of elephants most likely is also dependent on somatosensory feedback to correct limb sequencing. However, the weighting of the respective sensory feedback mechanisms remains to be explored.

Regardless, total neuromuscular delay in responding to perturbations scales [40] as body mass^0.21^, and an approximation of the time available to avoid a fall scales via pendulum period (limb length^0.5^; i.e. body mass^0.17^). Thus the delay relative to fall avoidance should scale with very slight positive allometry ∼body mass^0.04^, so at the very large size of elephants, this delay (and errors in control) could become a crucial constraint.

Despite recent research activity, wide gaps in the fundamental understanding of elephant locomotion remain. This knowledge gap impairs our understanding of how elephants control their locomotor patterns and more generally how such control scales with body size. Additionally, this gap hinders our ability to identify elephants that are moving abnormally due to health reasons. Stride time variability measures are a good indicator of poor neuromuscular health and possibly a propensity of falls in humans [18]. They also may be useful for the identification of neuromuscular pathologies including ataxia or arthritis in elephants. Less consistency in an elephant's walking pattern might be an indicator of lameness.

## 4. Conclusion

Visual feedback is necessary for correcting errors that may occur in the timings of Asian elephant locomotor patterns. An inability to properly integrate the available visual feedback may result in less consistency in the stride-to-stride sequencing of an elephant's limbs during walking. If these inconsistencies grow sufficiently large, they may result in a stumble or fall. Our results are the first to show that visual sensory feedback might play a role in the control of Asian elephants' uniquely specialized ‘graviportal’ locomotor mechanics, and should inspire more exploration of how variation influences the scaling of locomotor neuromechanics.

## Data Availability

Data are available from the Dryad Digital Repository: https://doi.org/10.5061/dryad.3j9kd51q3 [41].
